# Comparative transcriptome analysis reveals the genetic basis underlying the biosynthesis of polysaccharides in *Hericium erinaceus*

**DOI:** 10.1186/s40529-019-0263-0

**Published:** 2019-07-30

**Authors:** Nan Zhang, Zongfu Tang, Jun Zhang, Xin Li, Ziqian Yang, Chun Yang, Zhaofeng Zhang, Zuoxi Huang

**Affiliations:** 1 College of Life Sciences, Neijiang Normal University, Neijiang, 641100 People’s Republic of China; 2 Key Laboratory of Regional Characteristic Agricultural Resources, Department of Education, Neijiang, 641100 People’s Republic of China

**Keywords:** *Hericium erinaceus*, RNA-Seq, Comparative transcriptome, Polysaccharide biosynthesis, Erinacines

## Abstract

**Background:**

*Hericium erinaceus*, also known as lion’s mane mushroom, is a widely distributed edible and medicinal fungus in Asian countries. *H. erinaceus* harbors diverse bioactive metabolites with anticancer, immunomodulating, anti-inflammatory, antimicrobial, antihypertensive, antidiabetic and neuroprotective properties. Although the chemical synthesis processes of these bioactive metabolites are known, the biosynthetic processes remain unknown.

**Results:**

In this study, we obtained the transcriptomes of six *H. erinaceus* strains using next-generation RNA sequencing and investigated the characteristics of the transcriptomes and biosynthesis of bioactive compounds, especially polysaccharides. The transcriptomes ranged in size from 46.58 to 58.14 Mb, with the number of unigenes ranging from 20,902 to 37,259 across the six *H. erinaceus* strains. Approximately 60% of the unigenes were successfully annotated by comparing sequences against different databases, including the nonredundant (NR), Gene Ontology (GO), Kyoto Encyclopedia of Genes and Genomes (KEGG), clusters of orthologous groups for eukaryotic complete genomes (KOG) and Swiss-Prot databases. Most of the transcripts were putatively involved in signal transduction, carbohydrate metabolism, translation, transport and catabolism, and amino acid metabolism. Genes involved in polysaccharide biosynthesis were identified, and these genes encoded phosphoglucomutase (PGM), glucose phosphate isomerase (PGI), UDP-glucose pyrophosphorylase (UGP), glycoside hydrolase family proteins, glycosyltransferase family proteins and other proteins. Moreover, the putative pathway for the intracellular polysaccharide biosynthesis of *H. erinaceus* was analyzed. Additionally, the open reading frames (ORFs) and simple sequence repeats (SSRs) were predicted from the transcriptome data of the six strains.

**Conclusions:**

Overall, the present study may facilitate the discovery of polysaccharide biosynthesis processes in *H. erinaceus* and provide useful information for exploring the secondary metabolites in other members of the Basidiomycetes genus.

**Electronic supplementary material:**

The online version of this article (10.1186/s40529-019-0263-0) contains supplementary material, which is available to authorized users.

## Background

*Hericium erinaceus*, considered a delicacy in China since ancient times, is a valuable edible mushroom and one of the “top four treasures”, together with cubilose, trepang and shark fins (Huang 2018). The growth of *H. erinaceus* is strongly influenced by environmental conditions, such as air circulation, light, temperature, humidity, and pH (Jiang et al. 2014), thus increasing the value of *H. erinaceus*. In 1959, the artificial cultivation of *H. erinaceus* was first reported in China (Huang 2018). *H. erinaceus* is generally a good source of nutrients and health-promoting compounds (Cohen et al. 2014; Feeney et al. 2014a; Feeney et al. 2014b). Therefore, *H. erinaceus* is popular in Asian countries for both culinary and medicinal purposes (Friedman 2015).

Due to its anticancer, immunomodulating, hypolipidemic, antioxidant, anti-inflammatory, antimicrobial, antihypertensive, antidiabetic and neuroprotective properties (Kim et al. 2011a, b, 2013; Khan et al. 2013), many studies on the chemical isolation and physiological functions of bioactive metabolites in *H. erinaceus* have been performed in recent years. Among the bioactive compounds in *H. erinaceus*, polysaccharides play a major role in the medicinal properties of *H. erinaceus* (Chen 2016). Guo et al. (2012) reported that *H. erinaceus* polysaccharides enhanced cellular immunity and enhanced T cell function inhibited by TGF-β1. Moreover, the study demonstrated that polysaccharides enhanced T cells and macrophages to accelerate antitumor effects (Wang et al. 2001). The crude water-soluble polysaccharides of *H. erinaceus* upregulated certain functional immunomodulating events mediated by activated macrophages, such as the production of nitric oxide (NO) and the expression of cytokines (IL-1β and TNF-β), which might be responsible for the anticancer properties of this mushroom (Lee et al. 2009). Additionally, the study revealed that polysaccharides can significantly reduce the blood glucose concentration and affect the serum triglyceride and total cholesterol contents (Wang et al. 2005). In general, *H. erinaceus* polysaccharides can improve immunity, provide antitumor, antiaging and other effects and have broad applications.

Although the pharmacological molecular mechanisms of bioactive compounds of *H. erinaceus* have been researched, knowledge of the pathway involved in the biosynthesis of bioactive metabolites is limited by a lack of research. Chen et al. (2017) sequenced the genome in the monokaryotic mycelium, dikaryotic mycelium and fruiting body of *H. erinaceus* to investigate the biosynthesis of bioactive secondary metabolites from *H. erinaceus*. Zeng et al. (2018) identified numerous proteins involved in terpenoid, polyketide and sterol biosynthesis by proteome analysis of *H. erinaceus*. These two studies successfully provided a theoretical basis for elucidating the synthesis of active components. However, these two studies did not predict genes or proteins involved in the biosynthesis of polysaccharides, which are the most important substance in *H. erinaceus.*

In the present study, six strains (*H. erinaceus* sample: HT-4903, GT-06, CC-02, PZH-05, TJH-03 and TD-04) from different regions of China were used for transcriptome sequencing to investigate the mechanism of *H. erinaceus* polysaccharide biosynthesis. The transcriptomes of the six strains were obtained by high-throughput sequencing on an Illumina platform. We identified a set of gene clusters associated with the biosynthesis of bioactive compounds, especially polysaccharide biosynthesis. A total of 13 genes involved in polysaccharide biosynthesis were identified, such as phosphoglucomutase (PGM), glucose phosphate isomerase (PGI) and UDP-glucose pyrophosphorylase (UGP), which are most important to polysaccharide production. Then, functional annotation, expression analysis, and open reading frame (ORF) and simple sequence repeat (SSR) predictions were performed to detect the characteristics of the transcriptome structure. Our study will provide insights into the biosynthetic pathways of bioactive compounds and will be very useful for improving compound production in *H. erinaceus.*

## Methods

### Origin of strains and culture conditions

**Fig. 1:**
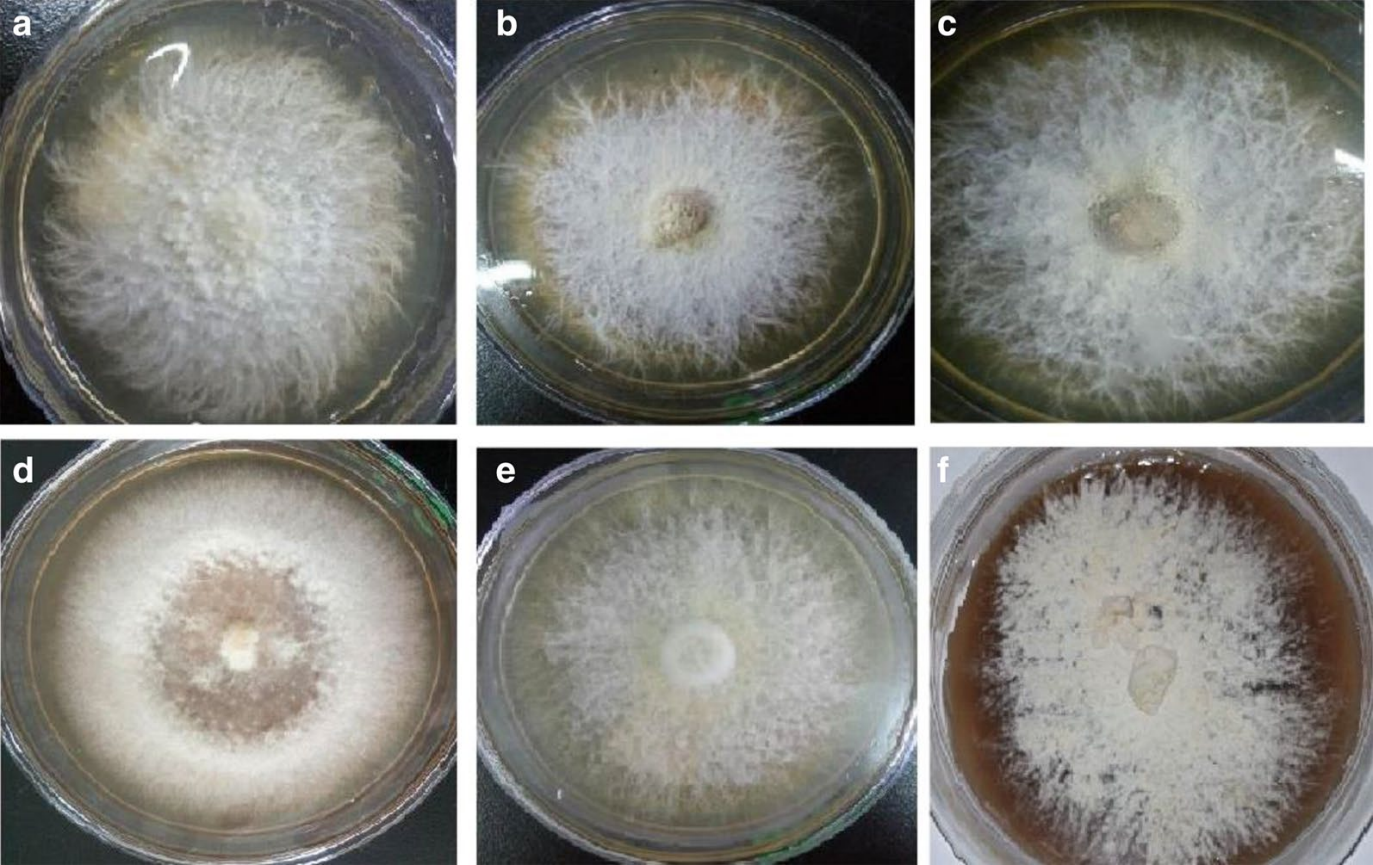
The morphological characteristics of *H. erinaceus* dikaryotic mycelium on PDA medium. **a** *H. erinaceus* HT-4903, **b** *H. erinaceus GT*-*06*, **c** *H. erinaceus* CC-02, **d** *H. erinaceus* PZH-05, **e** *H. erinaceus* TJH-03, **f** *H. erinaceus* TD-04

The haploid monokaryotic strains of the *H. erinaceus* samples included *H. erinaceus* HT-4903, *H. erinaceus* CC-02 (purchased from the Jiang du tian da Institute of Edible Fungi, Jiangsu, China), *H. erinaceus* GT-06 (Fujian, China), *H. erinaceus* PZH-05 (Sichuan, China), *H. erinaceus* TJH-03 (Sichuan, China), and *H. erinaceus* TD-04 (Hubei, China). Among these strains, *Hericium erinaceus* PZH-05 is a mutant strain and is mainly used for liquid fermentation processes. All of these strains were grown on potato dextrose agar (PDA) at room temperature for 3 weeks in darkness. The morphological characteristics of the six *H. erinaceus* strain samples are shown in Fig. 1 and Additional file 1: Table S1. In the third week of growth, mycelium samples were collected by scraping the top of the medium, and the samples were immediately frozen in liquid nitrogen and then stored at − 80 °C for total RNA extraction. Three biological replicates were performed for each *H. erinaceus* strain.

### Estimation of polysaccharide in *H. erinaceus*

The mycelium polysaccharides were extracted from the six *H. erinaceus* samples at the third week of growth. The mycelium obtained from each sample was dried after being scraped from the plates of the six strains. The dried mycelium (5 g) after extraction from each sample was further ground into a powder and resuspended in 20 volumes of water at 70 °C for 12 h. The supernatant was collected by centrifugation, concentrated by evaporation under reduced pressure, precipitated with 95% (v/v) ethanol to reach a final concentration of 80% (v/v) and then incubated at 4 °C for 12 h. The crude polysaccharides of each sample were obtained after centrifuging (4390×*g*, 20 min) and vacuum-drying (40 °C) the precipitate. The polysaccharide content was measured by the phenol-sulfuric acid method using glucose as a standard.

### Library construction and RNA sequencing

Total RNA from each sample was isolated using the RNAprep Pure Plant Kit (Bio TeKe, China) following the manufacturer’s instructions. The purity and concentration of RNA were determined using a NanoDrop-2000 spectrophotometer (Thermo Scientific, USA). Equal amounts of RNA from each sample that belonged to the same strain were pooled for cDNA library construction. Stranded cDNA libraries were constructed using the NEBNext Ultra Directional RNA Library Prep Kit (cat#E7420, NEB, UK) according to the manufacturer’s protocols. Briefly, mRNA was fragmented into 250–450 bp, followed by first-strand cDNA synthesis. Then, dUTP was added as a marker during the synthesis of the second-strand cDNA. Finally, the double-strand cDNA was digested with uracil—DNA glycocasylase (UDG) before the PCR. Thus, only the first strands of cDNA were retained and sequenced. Transcriptome sequencing was carried out on the HiSeq4000 (Illumina) platform using a paired-end run (2 × 150 bp).

### Transcriptome assembly and annotation

Raw reads were filtered by removing the adaptor sequences, reads with quality lower than Q20 and reads with poly-N. De novo transcriptome assembly for each strain was performed with Trinity software (Version 2.2.0) with default parameters (fixed k-mer value of 25) (Zhao et al. 2011). The expression levels of transcripts were normalized by calculating the fragments per kilobase of exon per million fragments mapped (FPKM) using RSEM software. The CDS and protein sequences of transcripts were predicted using TransDecoder (http://transdecoder.github.io/). The protein sequences were aligned to the four public databases: NCBI nonredundant protein sequences (NR; https://www.ncbi.nlm.nih.gov/refseq/about/nonredundantproteins/), Eukaryotic Orthologous Groups (KOG; https://genome.jgi.doe.gov/Tutorial/tutorial/kog.html), Swiss-Prot (a manually annotated and reviewed protein sequence database: http://www.ebi.ac.uk/uniprot), and Kyoto Encyclopedia of Genes and Genomes (KEGG) Orthology (KO; https://www.kegg.jp/kegg/ko.html) for functional annotation using the BLASTP program (cut-off E-value < 1 × 10^−5^). The Gene Ontology (GO; http://geneontology.org/) annotation of the proteins was carried out using WEGO software based on the NR annotation (Ye et al. 2006). The SSRs were detected using MIcroSAtellite identification tool (MISA) software (version 1.0). The minimum repeat numbers for motifs of mono-, di-, tri-, tetra-, penta-, and hexanucleotides were set as 10, 6, 5, 5, 5, and 5, respectively.

### Prediction of genes involved in polysaccharide biosynthesis in *H. erinaceus*

A BLAST search was performed for the prediction of genes participating in polysaccharide biosynthesis. Then, the genes involved in sucrose, fructose, mannose, and galactose metabolism and shared across the six strains of *H. erinaceus* were detected by manual processing. According to the NR database and KEGG annotation, the genes involved in the polysaccharide metabolism pathway were obtained. Additionally, the important genes involved in polysaccharide biosynthesis reported in a previous study were analyzed through the NR annotation results.

Statistical analyses were performed with Excel (2016). All of the data are expressed as the means and standard deviations of three replications.

### Quantitative reverse transcriptase-PCR (qRT-PCR)

The HiScript^®^ II Q RT SuperMix for qPCR (+g DNA wiper) Kit (Nanjing, China) was used according to the manufacturer’s instructions to generate the first-strand cDNA after extracting total RNA from six samples subjected to RNA-Seq. Ten genes were selected to validate the reliability of the RNA-Seq data. The gene-specific primers were produced by Primer 5.0 (Additional file 2: Dataset 1) and synthesized by Sangon Biotech (Shanghai) Co., Ltd. (Shanghai, China). ChamQTM SYBR^®^ Color qPCR Master Mix (10 μL; Vazyme, Nanjing, China) was mixed with the gene-specific primers, sterilized water and the synthesized cDNA in a total reaction volume of 20 μL. Reactions were performed on a qTOWER 2.2 (Analytik Jena AG, Jena, Germany). The two-step quantitative RT-PCR program was performed at 95 °C for 30 s, followed by 40 cycles of 95 °C for 10 s and 60 °C for 30 s. The 2^−∆∆Ct^ method was used to calculate the relative expression level of each gene, and actin was selected as the reference gene for normalization (Livak and Schmittgen 2001). Each reaction was carried out with three biological replicates and three technical replicates.

## Results

### Illumina sequencing and de novo assembly

Raw data were generated by sequencing each *H. erinaceus* strain. After the reads were filtered and subjected to quality control, a total of 21 to 46 million clean reads were obtained for *H. erinaceus* HT-4903, GT-06, CC-02, PZH-05, TJH-03 and TD-04 (Table 1). The quality of most bases along the reads were above Q30, and more than 96% of the reads had a quality score > Q30 (Table 1 and Additional file 1: Fig. S1). The contents of bases A and T were very similar, as well as the C and G contents, suggesting a balance among bases across the reads (Additional file 1: Fig. S2). These results suggested that the clean reads with high quality could be used for subsequent analyses.

**Table 1:** Summary of the sequencing and assembly of six *H. erinaceus* strain samples

Sequencing index	HT-4903	GT-06	CC-02	PZH-05	TJH-03	TD-04
Total number of clean reads	28,172,192	44,081,115	24,313,274	21,587,820	22,681,715	46,117,401
Total number of clean bases (Gb)	8.32	13.06	7.15	6.36	6.67	13.52
GC content (%)	56.77	56.62	56.51	57	56.13	53.36
Q30 content (%)	97.21	97.07	97.24	96.77	97.13	97.28
Total number of transcripts	36,945	40,141	36,065	25,905	47,294	40,590
N50 value of transcript (bp)	2579	2220	2470	2946	1991	1990
Total bases of transcript (Mb)	58.14	55.24	55.51	46.58	53.11	47.69
Total number of unigene	22,618	24,915	22,284	20,902	37,259	28,640
Median length of unigene (bp)	549	498	574	1012	450	438
N50 value of unigene (bp)	2195	1944	2111	2431	1668	1708
Total bases of unigene (Mb)	26,13	25,66	25,47	31	33,54	25,82

Then, all clean reads in each strain were de novo assembled into 36,945, 40,141, 36,065, 25,905, 47,294 and 40,590 transcripts for *H. erinaceus* HT-4903, GT-06, CC-02, PZH-05, TJH-03 and TD-04 using Trinity, respectively (Table 1). The size of these transcripts ranged from 46.58 Mb in *H. erinaceus* PZH-05 to 58.14 Mb in *H. erinaceus* HT-4903. The N50 values of transcripts in the six strains were 2579 bp, 2220 bp, 2470 bp, 2946 bp, 1991 bp and 1990 bp, respectively. Then, the longest transcript of each gene was used as a unigene. After the redundant transcripts were removed, 22,618, 24,915, 22,284, 20,902, 37,259 and 28,640 unigenes with N50 values of 2195 bp, 1944 bp, 2111 bp, 2431 bp, 1668 bp and 1708 bp were obtained for *H. erinaceus* HT-4903, GT-06, CC-02, PZH-05, TJH-03 and TD-04, respectively (Table 1).

### Functional annotation of the transcripts

Functional annotation of the predicted genes was performed using BLAST (Altschul et al. 1990) against the following six databases: GO (Ashburner et al. 2000), KEGG (Kanehisa et al. 2016), KOG (Tatusov et al. 2003), Swiss-Prot (Gasteiger et al. 2001) and NCBI NR protein databases. A total of 19.01–65.98% of transcripts returned a BLAST hit above the E-value cut-off of 10^−5^ (E-value < 1 × 10^−5^) from these five databases (Table 2). Among these transcripts, only 8729–16,622 transcripts (33.70–40.95% of the total) were not matched with these databases (Table 2). Most of the transcripts in six strains were successfully annotated in each database.

**Table 2:** Functional annotations of the de novo transcriptomes for HT-4903, GT-06, CC-02, PZH-05, TJH-03 and TD-04

Database	HT-4903	GT-06	CC-02	PZH-05	TJH-03	TD-04
NR	23,116 (62.57%)	24,517 (61.08%)	22,760 (63.11%)	17,093 (65.98%)	30,950 (65.44%)	23,399 (57.65%)
GO	15,162 (41.04%)	15,780 (39.31%)	14,917 (41.36%)	11,382 (43.94%)	20,775 (43.93%)	14,755 (36.35%)
KO	8662 (23.45%)	8893 (22.15%)	8489 (23.54%)	6004 (23.18%)	11,673 (24.68%)	8668 (21.36%)
KOG	9299 (25.17%)	9210 (22.94%)	9085 (25.19%)	7196 (27.78%)	11,383 (24.07%)	8489 (20.91%)
Swiss-Prot	7099 (19.22%)	7631 (19.01%)	7118 (19.74%)	7798 (30.10%)	12,695 (26.84%)	8353 (20.58%)
Unannotated	13,723 (37.14%)	15,422 (38.42%)	13,239 (36.71%)	8729 (33.70%)	16,256 (34.37%)	16,622 (40.95%)
Total	36,945 (100.00%)	40,141 (100.00%)	36,065 (100.00%)	25,905 (100.00%)	47,294 (100.00%)	40,590 (100.00%)

**Fig. 2:**
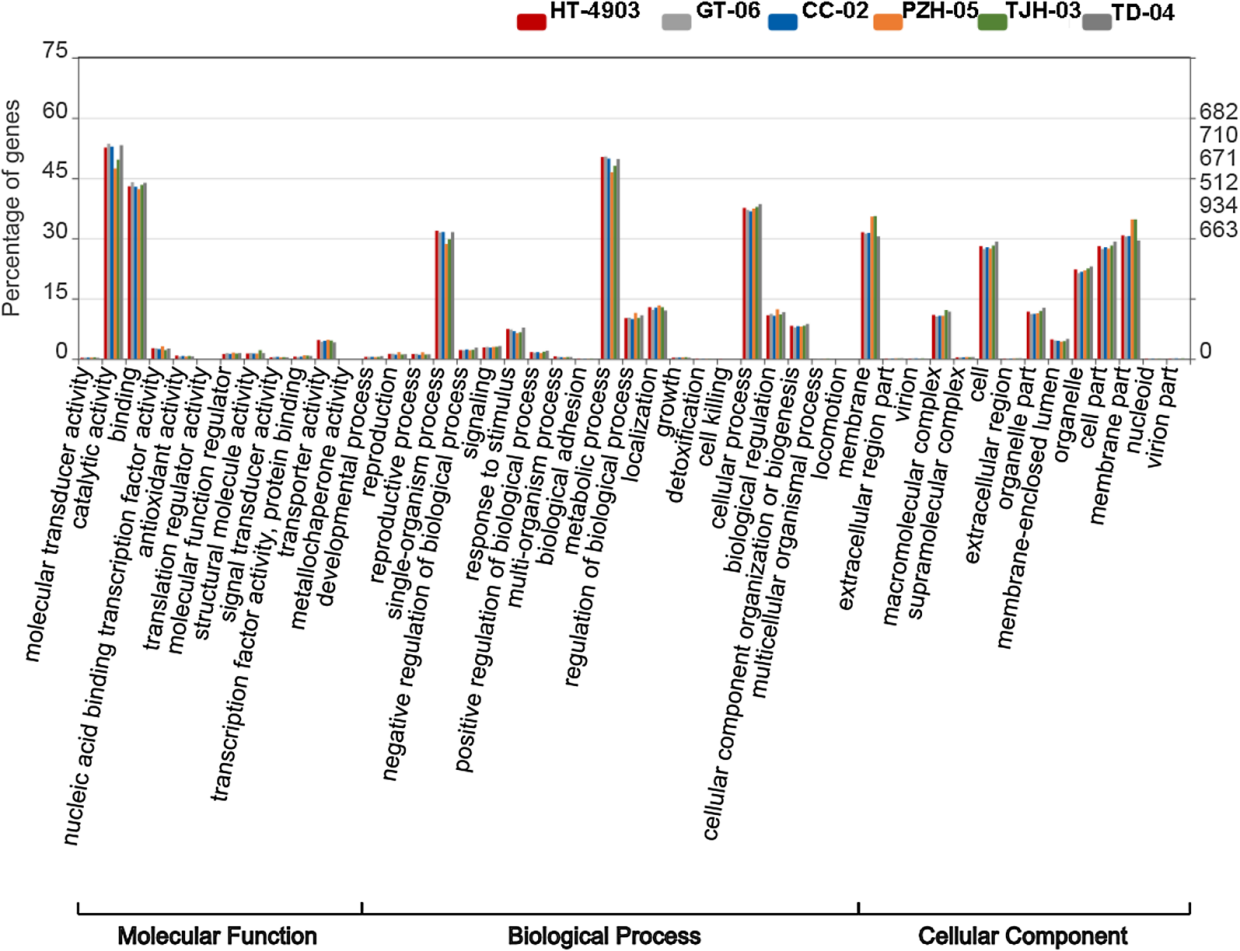
GO classification of unigenes in the six *H. erinaceus* strains

**Fig. 3:**
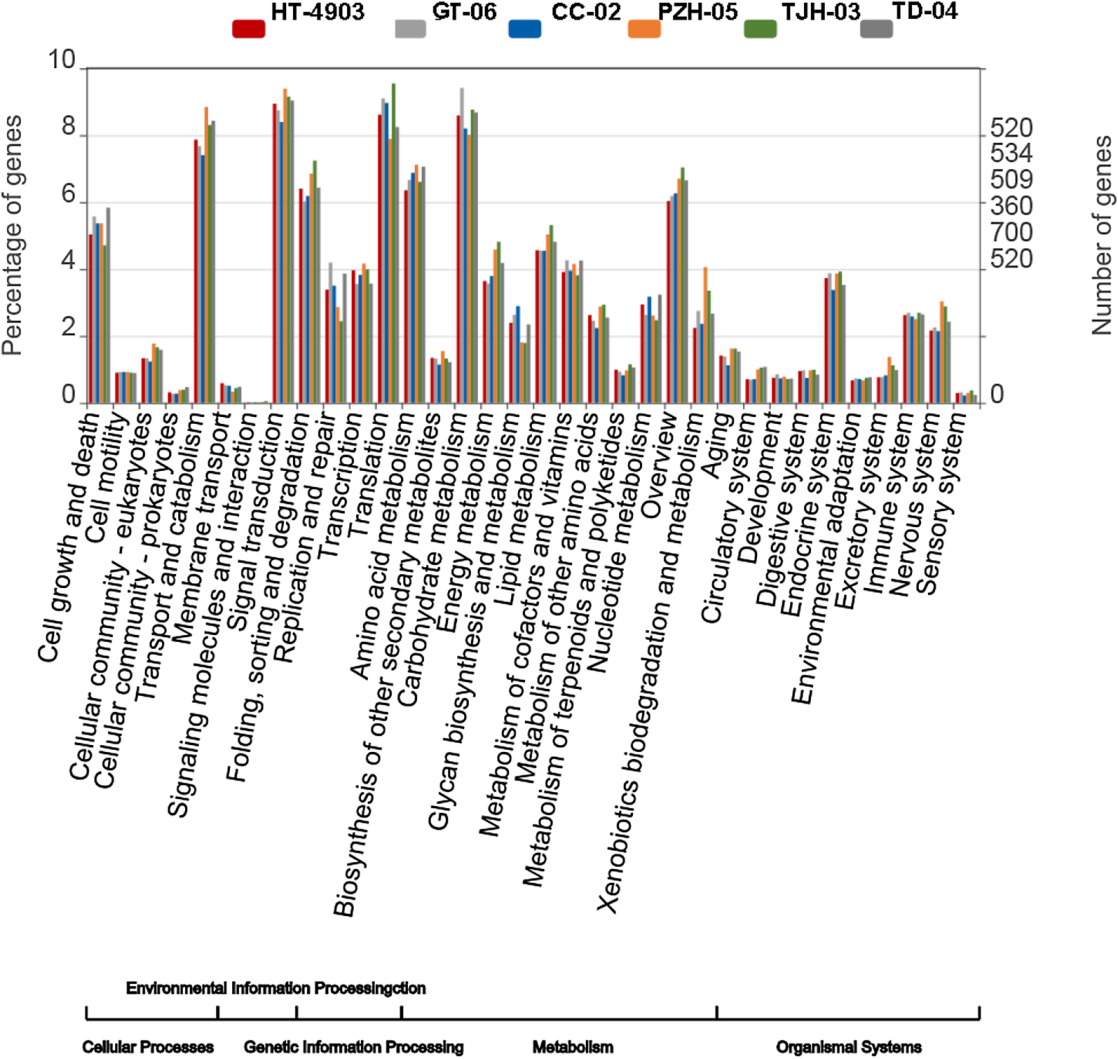
KEGG pathway classification of unigenes in the six *H. erinaceus* strains

Then, we obtained the GO classification using these transcripts. Intriguingly, the distribution of annotated genes at the level-two GO terms in the six strains showed highly similar patterns (Fig. 2). In the GO classification, all of the genes annotated in the GO database were classified into three main categories and contained 46 level-two GO terms. The top five clustered classes in function were catalytic activity, metabolic process, binding, cellular process and membrane. The percentage of annotated genes in the top five clusters was more than 30% (Fig. 2). These highly enriched GO terms mainly referred to the maintenance of the basic regulation and metabolic functions of the six strains. Additionally, we performed KEGG pathway analysis to understand the biological functions and interactions among gene products. A total of 36 pathways in 5 categories were retrieved. In the KEGG pathway analysis, the top five clustered classes were signal transduction, carbohydrate metabolism, translation, transport and catabolism, and amino acid metabolism (Fig. 3).

### Analysis of expression level and prediction ORFs and SSRs in transcripts

We used TransDecoder and MISA to predict the ORFs and SSRs of the transcripts in the six strains. The average number of ORFs was 24,215, ranging from 18,292 in *H. erinaceus* PZH-05 to 29,125 in *H. erinaceus* TJH-03 (Table 3). Then, a search of SSR loci was conducted for these transcripts. The number of transcripts containing SSR sites in each of the six strains was 3691, 6278, 3762, 3770, 4638 and 5302, accounting for 10–17% of the total transcripts. After statistical analysis, we found that the transcriptomes of the six *H. erinaceus* strains were most abundant in mono-, di-, tri-, quad-, penta-, hexanucleotide repeat motifs. Moreover, the proportion of repeat types varied greatly (Table 4). Among them, mononucleotide, dinucleotide and trinucleotide motifs were the predominant repeat motifs. The number of mononucleotide SSRs was the largest (2715–5407), accounting for 68.28–82.47%, followed by dinucleotides (488–1008) and trinucleotides (157–236), accounting for 11.93–25.35 and 2.87–5.77%, respectively.

**Table 3:** The number of predicted ORFs in six *H. erinaceus* transcripts

Categories	HT-4903	GT-06	CC-02	PZH-05	TJH-03	TD-04
Transcripts with predicted ORFs	24,398 (66.04%)	25,512 (63.56%)	24,241 (67.21%)	18,292 (70.61%)	29,125 (61.58%)	23,727 (58.46%)
Total Transcripts	36,945	40,141	36,065	25,905	47,294	40,590

**Table 4:** Distribution of SSR loci in *H. erinaceus* HT-4903, GT-06, CC-02, PZH-05, TJH-03 and TD-04 transcriptome

SSR type	Sample					
	HT-4903	GT-06	CC-02	PZH-05	TJH-03	TD-04
Mono-nucleotide	2715 (68.28%)	5407 (80.89%)	2811 (69.27%)	3331 (81.44%)	4014 (82.47%)	4495 (78.92%)
Di-nucleotide	157 (3.95%)	192 (2.87%)	178 (4.39%)	236 (5.77%)	175 (3.60%)	214 (3.76%)
Tri-nucleotide	1008 (25.35%)	992 (14.84%)	984 (24.24%)	488 (11.93%)	657 (13.50%)	898 (15.77%)
Quad-nucleotide	64 (1.61%)	68 (1.01%)	54 (1.33%)	22 (0.54%)	8 (0.16%)	62 (1.09%)
Penta-nucleotide	12 (0.3%)	9 (0.13%)	13 (0.32%)	11 (0.27%)	2 (0.04%)	6 (0.11%)
Hexa-nucleotide	20 (0.5%)	16 (0.23%)	18 (0.44%)	2 (0.05%)	11 (0.22%)	21 (0.37%)
Total	3976	6684	4058	4090	4867	5696

To quantify the expression levels of the unigenes, the Bowtie 2 (Langmead 2010) program was applied using RSEM (Li and Dewey 2011). Then, we obtained the number of mapped reads and separately calculated the FPKM values and ranked the values from high to low from the data of the six strains. The statistical results of gene and transcript expression levels (FPKM) in the six samples are shown in Additional file 1: Table S2. Among the six strains, the proportion of genes and transcripts in different FPKM intervals was similar. The FPKM distribution of the six samples is shown in Additional file 1: Figs. S3 and S4.

Among all FPKM values, we selected the top 10 FPKM values from the six strains to investigate the highly expressed genes (Additional file 3: Dataset 2). The GO annotation revealed that these genes were mainly involved in oxidation-reduction processes, translation, catalytic activity, ATP binding, and protein ubiquitination.

### Comparison of polysaccharide metabolism-related gene expression in six strains

**Fig. 4:**
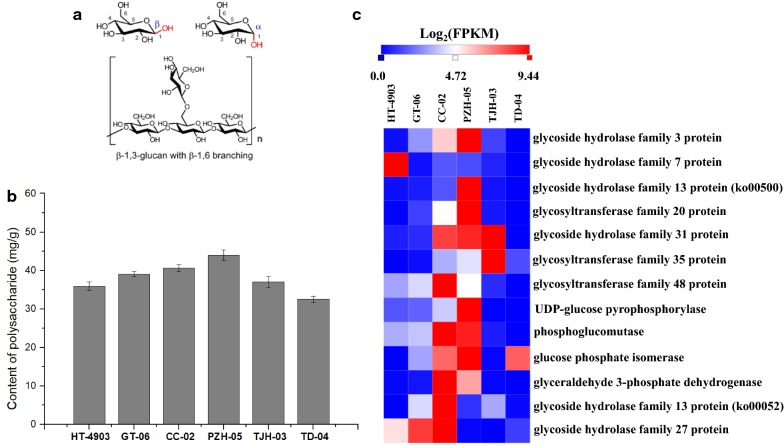
The basic structure of typical mushroom polysaccharides (**a**), polysaccharide content (**b**) and the expression of important genes for polysaccharide biosynthesis (**c**). Mean polysaccharide contents are shown with standard errors bars from three repeated experiments

Polysaccharides are considered the major and most-studied active component in *H. erinaceus* (Lu et al. 2014). *H. erinaceus* polysaccharides are polymers composed of more than 10 monosaccharides that exist in mycelia, fruiting bodies and fermentation broth (Zhang et al. 2016). The basic structure of a typical mushroom polysaccharide is shown in Fig. 4a (Friedman 2016). In this study, we measured the polysaccharide contents in the six strains. As shown in Fig. 4b, the mycelium of strain PZH-05 contained more polysaccharides than other strains. The polysaccharide content in *H. erinaceus* PZH-05 reached 43.97 mg/g, which was the highest polysaccharide content detected among the strains, followed by *H. erinaceus* CC-02 (40.63 mg/g), GT-06 (39.07 mg/g), TJH-03 (37.02 mg/g), HT-4903 (35.90 mg/g), and TD-04 (32.57 mg/g). The polysaccharide levels in *H. erinaceus* PZH-05 were 0.74 times higher than that in TD-04.

Recently, many various polysaccharides have been extracted from fungi of the *Hericium* genus, and research has revealed that polysaccharides are mainly composed of monosaccharides, such as glucose, galactose, mannose, arabinose and xylose, as well as rhamnose, fucose and fucose (Jia et al. 2004; Wang et al. 2004). Therefore, to investigate the polysaccharide biosynthesis pathway of *H. erinaceus*, we detected the genes and gene clusters involved in the saccharide unit, such as sucrose, mannose and galactose biosynthesis. A comparison of genes involved in polysaccharide biosynthesis in the six strains was then conducted. A total of 13 genes involved in saccharide unit metabolism was shared among the six strains (Fig. 4c). Among these encoding genes, glycoside hydrolase family 3 protein, glycoside hydrolase family 13 protein and glycoside hydrolase family 31 protein are the key enzymes for catalyzing the biosynthesis of d-glucose. The genes encoding glycoside hydrolase family 7 protein, glycosyltransferase family 20 protein, glycosyltransferase family 35 protein and glycosyltransferase family 48 protein catalyze the biosynthesis of cellobiose, trehalose-6P, α-d-glucose-1P and 1,3-β-glucan, respectively. All of these genes were highly expressed (FPKM value > 1), and the expression patterns were similar (Fig. 4c). Most genes had the highest FPKM value in *H. erinaceus* PZH-05, followed by *H. erinaceus* CC-02, GT-06, TJH-03, HT-4903, and TD-04. This result paralleled the physiological data and coincided with the trend in polysaccharide content in the six strains.

### Genes involved in starch and sucrose metabolism

We collected the transcripts involved in sucrose metabolism using KEGG annotation. There are 59, 69, 65, 43, 94 and 64 genes annotated in starch and sucrose metabolism (ko00500) for *H. erinaceus* HT-4903, GT-06, CC-02, PZH-05, TJH-03 and TD-04, respectively (Additional file 1: Table S3). Among them, 7 genes were shared among the six strains (Table 5). Figure 5a shows the pathways in which these 7 genes are involved. Genes were identified that encode glycoside hydrolase family 3 protein, glycoside hydrolase family 13 protein and glycosyltransferase family 20 protein, which are the key enzymes for the biosynthesis of d-glucose, and the gene encoding glycosyltransferase family 48 protein was identified, which is an important enzyme for 1,3-β-glucan synthases. The expression level data indicated that the 7 genes were highly expressed in the six strains, and the maximum FPKM value reached 257.86 in the strain *H. erinaceus* PZH-05 (Additional file 4: Dataset 3).

**Table 5:** Genes and enzymes involved in *H.einaceus* polysaccharide metabolism in six strains

KEGG_map	No.	NR_defination	FPKM_value					
			HT-4903	GT-06	CC-02	PZH-05	TJH-03	TD-04
ko00500	1	Glycoside hydrolase family 3 protein	6.91	19.74	34.52	53.9	11.72	5.61
	2	Glycoside hydrolase family 7 protein	8.76	2.64	3.54	3.40	2.88	2.46
	3	Glycoside hydrolase family 13 protein	17.74	23.99	51.36	257.86	19.78	10.91
	4	Glycosyltransferase family 20 protein	6.17	9.49	19.96	33.47	7.24	6.08
	5	Glycoside hydrolase family 31 protein	4.27	5.34	40.8	43.25	46.46	1.69
	6	Glycosyltransferase family 35 protein	43.3	45.35	73.97	82.88	132.89	56.81
	7	Glycosyltransferase family 48 protein	44.28	51.7	93.56	57.51	27.16	21.06
ko00051	1	Glycoside hydrolase family 5 protein	74.7	80.59	166.69	none	6.44	53.15
	2	Fructose-bisphosphate aldolase	107.64	86.32	110.04	none	3.84	64.18
	3	Bifunctional 6-phosphofructo-2-kinase/fructose-2,6-bisphosphate 2-phosphatase	46.85	47.26	51.20	none	18.29	30.21
	4	DAK1/DegV-like protein	10.44	2.39	2.93	none	1.50	3.11
	5	Dihydroxyacetone kinase 1	5.40	8.35	8.84	none	1.68	7.26
ko00052	1	Glycoside hydrolase family 13 protein	3.64	23.99	51.36	8.15	19.31	4.35
	2	Glycoside hydrolase family 27 protein	128.13	199.79	224.94	5.69	3.16	29.58
	3	Glycoside hydrolase family 31 protein	4.27	5.34	40.8	43.25	46.46	1.69
Key enzyme	1	UDP-glucose pyrophosphorylase,	19.12	21.4	39.38	91.81	5.04	4.83
	2	Phosphoglucomutase	67.65	70.63	115.33	111.17	47.07	43.07
	3	Glucose phosphate isomerase	1.91	24.45	58.06	72.04	2.42	59.11

#FPKM value is the mean expression value of three biological replicates. NR_defination means the protein annotated result of six strains transcripts in NR database

**Fig. 5:**
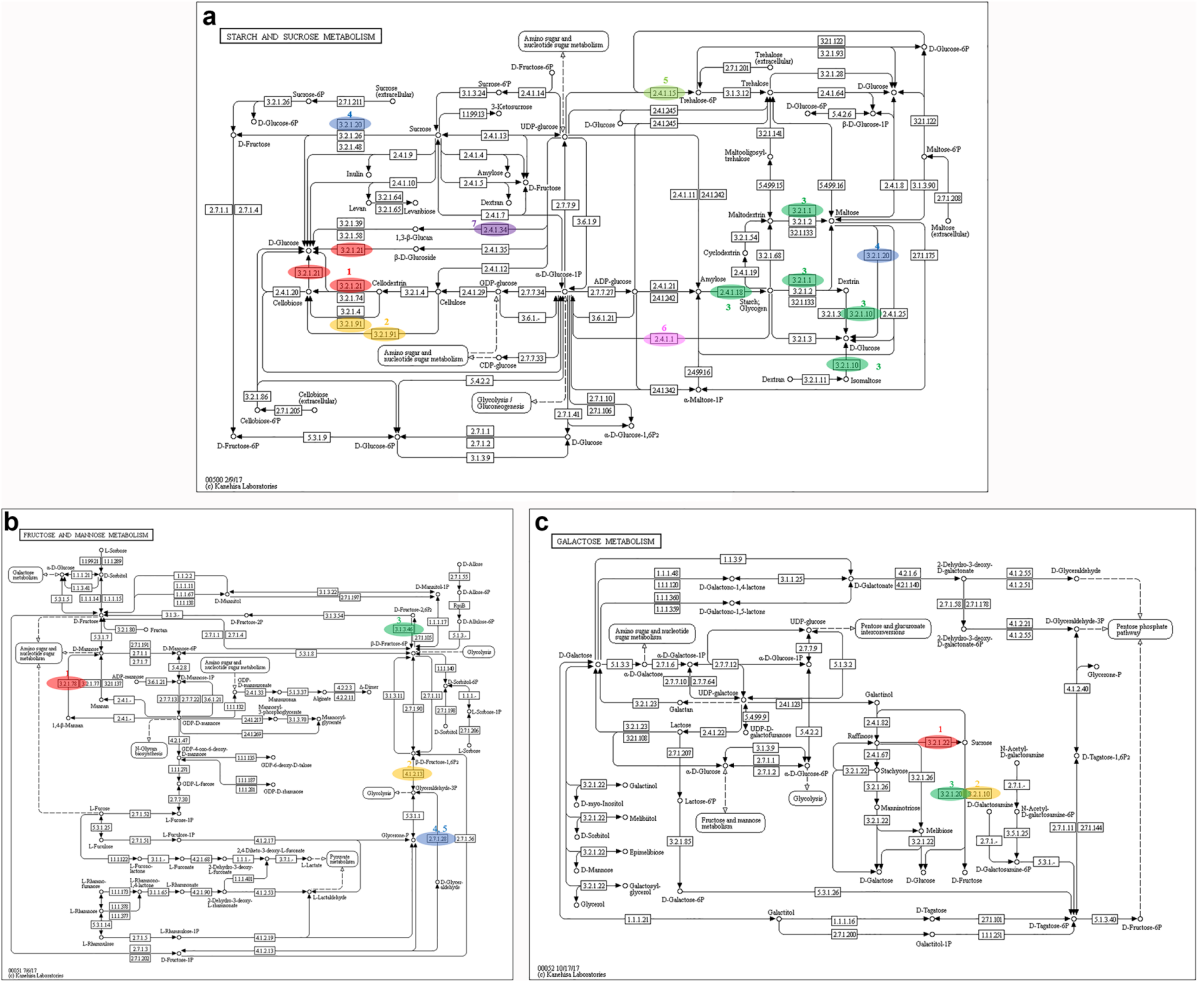
KEGG mapping of the polysaccharide metabolism pathway identified in *H. erinaceus*. **a** KEGG map 00500, **b** KEGG map 00051, and **c** KEGG map 00052. The colorized oval in the map indicates the related genes from our data in this pathway. The number beside the oval is in accordance with the gene number in Table 5. Each color indicates different genes. The same color indicates the same genes

### Genes involved in fructose and mannose metabolism

The genes involved in fructose and mannose biosynthesis were identified, and a total of 5 genes that were involved in this pathway were identified across all strains except *H. erinaceus* PZH-05 (Table 5). These five genes encoded glycoside hydrolase family 5 protein, fructose-bisphosphate aldolase, bifunctional 6-phosphofructo-2-kinase/fructose-2,6-bisphosphate 2-phosphatase, DAK1/DegV-like protein and dihydroxyacetone kinase 1. As shown in Fig. 5b, these genes encode the key enzymes for d-mannose, d-fructose-2,6 P2, β-d-fructose-1,6 P2 and glyceraldehyde-3P biosynthesis. The gene encoding glycoside hydrolase family 5 protein had low expression level in *H. erinaceus* TJH-03 (FPKM value equal 6.44) but was highly expressed in the other 4 strains, with FPKM values greater than 50 (Additional file 4: Dataset 3). A similar pattern of expression levels, in which expression was slightly lower in the *H. erinaceus* TJH-03 strain but highly expressed in the other 4 strains, was detected for the 4 genes encoding fructose-bisphosphate aldolase, bifunctional 6-phosphofructo-2-kinase/fructose-2,6-bisphosphate 2-phosphatase, DAK1/DegV-like protein and dihydroxyacetone kinase 1 (Additional file 4: Dataset 3).

### Genes involved in galactose metabolism

We obtained 3 genes shared across the six strains enriched in the galactose metabolism pathway (ko00052) (Table 5). Although these 3 annotated genes occurred in galactose metabolism, the main function of the 3 encoding genes is sucrose, d-glucose and d-fructose biosynthesis (Fig. 5c). Among them, the gene encoding glycoside hydrolase family 27 protein was relatively highly expressed in the six *H. erinaceus* strains (Additional file 4: Dataset 3).

We also collected genes involved in terpenoid biosynthesis in the KEGG pathway, such as the encoding genes of mevalonate pyrophosphate decarboxylase, terpenoid synthase, and 3-hydroxy-3-methylglutaryl-coenzyme A reductase. These genes were also highly expressed in the six strains (Additional file 5: Dataset 4). Interestingly, Chen also predicted the gene encoding 3-hydroxy-3-methylglutaryl-coenzyme A reductase and found that these genes were highly expressed in the monokaryotic mycelium, dikaryotic mycelium and fruiting body of *H. erinaceus* (Chen et al. 2017). Similarly, we obtained this expected protein, and KEGG annotation revealed that these genes participated in terpenoid backbone biosynthesis (ko00900) and ubiquinone and other terpenoid-quinone biosynthesis (ko00130). We predicted that these genes also play an essential role in the biosynthesis of terpenoids.

### Predicting the molecular mechanism of *H. erinaceus* polysaccharide biosynthesis

**Fig. 6:**
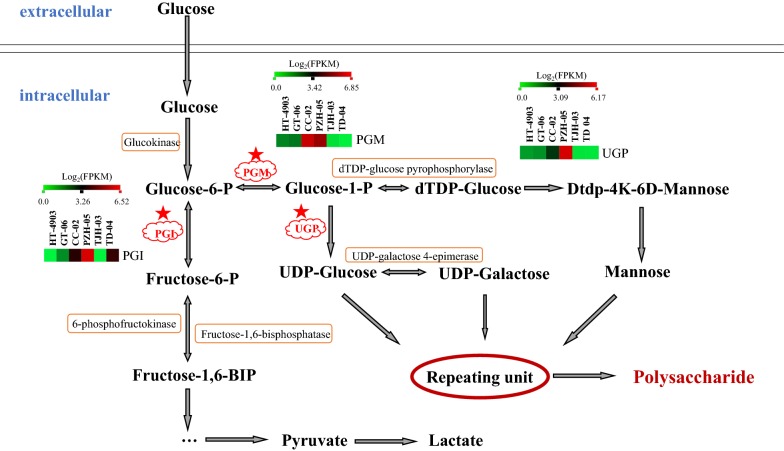
Putative pathway for intercellular polysaccharide biosynthesis in *H. erinaceus*. The red stars indicate the three most important enzymes involved in polysaccharides

*H. erinaceus* polysaccharides are glucans that are composed of main chains connected by β-(1,3) bonds and branched chains connected by β-(1,6) bonds (Fan and Huang 2008). Currently, due to the complexity of fungal secondary metabolite synthesis, research on polysaccharide biosynthesis is mainly focused on bacteria and rarely on fungi (Freitas et al. 2011). Existing studies have shown that the process of the biosynthesis of polysaccharides is conserved, although the structure of polysaccharides varies substantially (Ruas-Madiedo et al. 2002). The biosynthesis of polysaccharides mainly includes the synthesis of precursor nucleotide sugars (activation of monosaccharides), extension and polymerization of repeating units, and output of polysaccharides (Levander and Radstrom 2001; Knirel and Valvano 2013). Figure 6 shows the partial pathway for intracellular polysaccharide biosynthesis. After glucose is converted to glucose-6 phosphate, there are two important metabolism branches, the fructose-6-P branch and the glucose-1-P branch. Therefore, the enzymes that catalyze these two branch reactions are most important in polysaccharide biosynthesis. The study also revealed that PGM, PGI and UGP were the key enzymes affecting the output and types of polysaccharides in *Ganoderma lucidum* (Liu et al. 2011). In our study, the genes of PGM, PGI and the UGP were identified by the homology sequence search method (BLAST), and the FPKM values were calculated for these three genes (Fig. 6). The results indicated that the expression trends of these 3 encoding genes were similar and had the highest value in PZH-05 (Table 5 and Fig. 6).

Research has shown that the β-glucans are overproduced by glyceraldehyde-3-phosphate regulation and that glyceraldehyde-3-phosphate is an important protein in polysaccharide biosynthesis (Chai et al. 2013). Additionally, UGP is regarded as a key enzyme involved in polysaccharide biosynthesis (Yan et al. 2017). We also analyzed the two encoding genes using previous transcriptome data. The results showed that the genes encoding glyceraldehyde-3-phosphate were highly expressed across the six strains (Additional file 5: Dataset 4). The KEGG annotation of this gene is ko04066 (HIF-1 signaling pathway), ko00010 (glycolysis/gluconeogenesis), ko00710 (carbon fixation in photosynthetic organisms), ko01230 (biosynthesis of amino acids) and ko01200 (carbon metabolism). Moreover, we found that the UGP gene that existed in 5 strains, excluding HT-4903, was expressed at lower levels than glyceraldehyde-3-phosphate (Additional file 5: Dataset 4).

### qRT-PCR validation

**Fig. 7:**
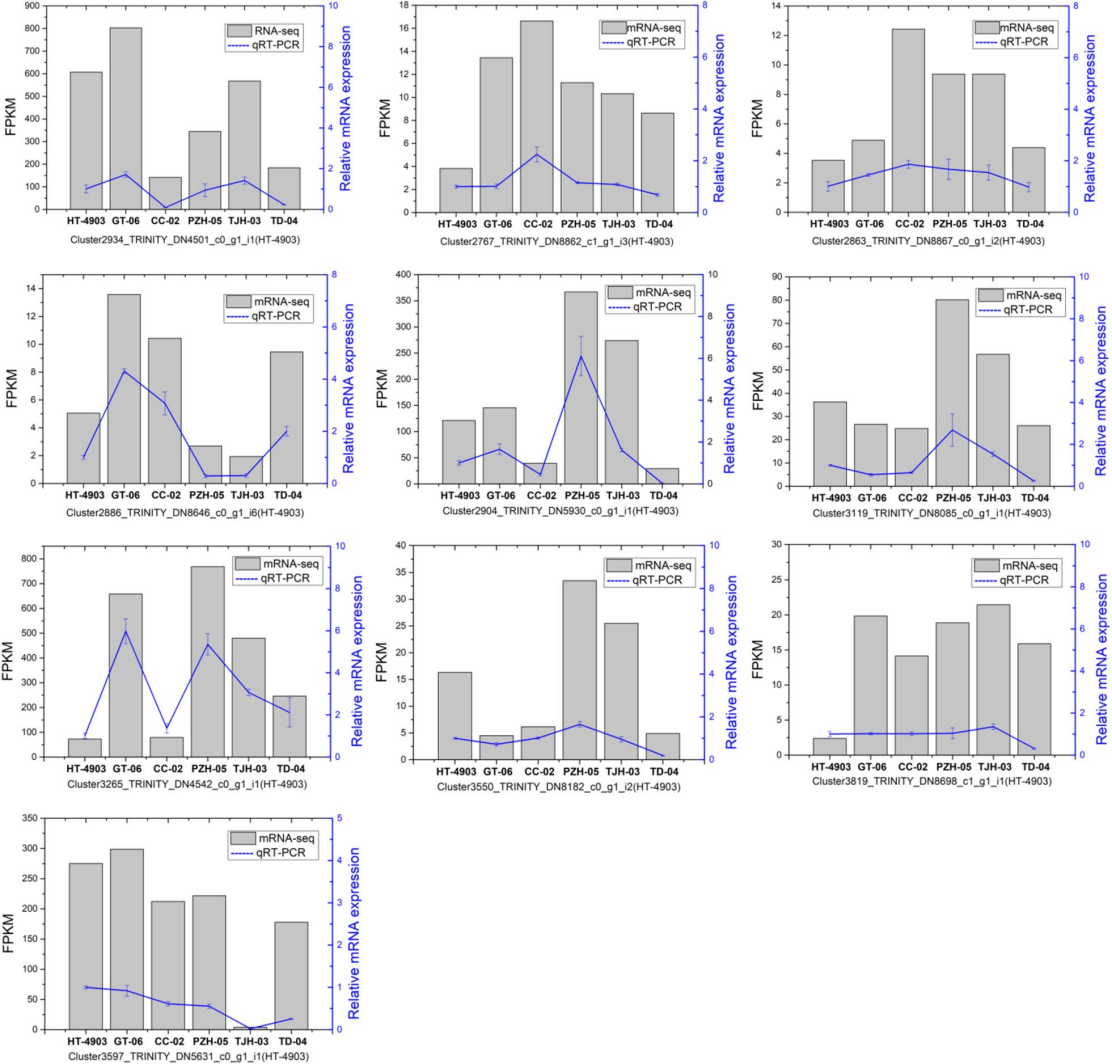
qRT-PCR confirmation of 10 expressed genes in the six *H. erinaceus* strains. The expression patterns of selected genes were analyzed across six samples. Gray bars with standard errors represent the FPKM values according to RNA-Seq (left y-axis), and blue lines indicate the relative expression level determined by qRT-PCR (right y-axis)

To verify the gene expression level produced by RNA-Seq, we performed qRT-PCR assays with six independent samples for RNA-Seq. We selected 10 genes with varying degrees of expression to validate the RNA-Seq data. Among them, 2 genes were involved in polysaccharide biosynthesis. As expected, these genes had similar expression tendencies. The qRT-PCR data for these genes were basically consistent with the RNA-Seq data (Fig. 7). This result indicates that the RNA-Seq data are accurate and valuable.

## Discussion

Research in medicine and pharmacology has indicaed proven that polysaccharides of *H. erinaceus* have certain therapeutic effects on improving immunity, restraining cancer and senescence, reducing blood sugar, and other effects (Illum 1998; Wang et al. 2001; Park et al. 2002; Fan and Huang 2008). Although many studies have unveiled the mechanism underlying the medicinal effect of *Hericium* polysaccharides, the complex properties of *H. erinaceus* polysaccharides and metabolic processes have limited the ability to study the biosynthesis mechanism of polysaccharides in *H. erinaceus*. However, the development of RNA-Seq technology has provided new opportunities for the study of the metabolic mechanism of *H. erinaceus* polysaccharides. RNA-Seq approaches enabled the comparative quantification of gene expression in different organisms and is used broadly across diverse organisms representing unique physiological processes. The advantages of this technology have enabled the identification of candidate genes involved in a variety of processes (Wang et al. 2009).

In our research, we carried out de novo transcriptome sequencing, assembly, and annotation of six *H. erinaceus* strains. Extensive transcriptome datasets of the six strains were generated. Moreover, functional annotation, expression patterns, and gene clusters involved in the *H. erinaceus* polysaccharide biosynthesis pathway were conducted. The shared genes across the six strains related to the synthesis of sucrose, galactose and mannose, which are the units of polysaccharide biosynthesis, were identified. Our results showed that these genes played an important role in d-glucose, d-mannose, and sucrose biosynthesis. Moreover, we found that the genes encoding UGP participated in sucrose synthases with high expression in the six strains. In addition, we mapped the putative pathway for intracellular polysaccharide biosynthesis of *H. erinaceus*. Our study showed that the genes of PGM, PGI and the UGP were highly expressed across the six strains, and the expression pattern was consistent with the polysaccharide content of the six strains. These results strongly indicate that PGM, PGI and UGP are key enzymes for *H. erinaceus* polysaccharide biosynthesis.

Interestingly, the genes involved in polysaccharide metabolism identified in *H. erinaceus* PZH-05 were significantly different from the other five strains of *H. erinaceus*. The phenotypic analysis showed that the *H. erinaceus* PZH-05 grew rapidly, had no aerial mycelia and formed a macroscopic fruit body under suitable conditions. On the one hand, we suspected that the differences in polysaccharide metabolism genes were related to the characteristics of *H. erinaceus* PZH-05; on the other hand, the mechanism of polysaccharide metabolism in *H. erinaceus* PZH-05 was different from the five other strains. The differences in polysaccharide synthesis and phenotypic characteristics of *H. erinaceus* PZH-05 remain to be further verified and studied.

In addition to the polysaccharide biosynthesis pathway, we also focused on the biosynthesis of erinacines. Our study concluded that 3-hydroxy-3-methylglutaryl-coenzyme A reductase, terpenoid synthase and mevalonate pyrophosphate decarboxylase are genes predicted to participate in erinacine biosynthesis. These three genes are highly expressed in the 6 strains, and the range of the FPKM value was 3.02–143.71. Interestingly, 3-hydroxy-3-methylglutaryl-coenzyme A reductase was predicted to be involved in terpenoid biosynthesis, which was consistent with the findings of Chen et al., who also suggested that this gene was highly expressed in three tissues of *H. erinaceus* (Chen et al. 2017).

According to the functional annotation, the transcripts that participated in signal transduction, carbohydrate metabolism, translation, transport and catabolism, and amino acid metabolism were largely enriched. At the level-two GO categories of molecular function, biological process and cellular component, most of the transcripts were classified into signal transduction, carbohydrate metabolism and translation, indicating the basic functions of transcripts. Furthermore, we also predicted the ORFs and SSRs in *H. erinaceus* transcripts. The results of the predicted ORFs and SSRs supply theoretical support for genetic diversity analyses, genetic linkage map construction and marker-assisted selection (Qiu et al. 2014; Nie et al. 2015; Guo et al. 2017; Lei et al. 2017).

## Conclusion

In summary, the analysis of the transcriptome sequences of six *H. erinaceus* strains via an Illumina platform was a powerful method for investigating the putative genes involved in diverse secondary metabolite biosynthesis. In our study, a total of 20,902–37,259 unigenes were obtained from the six *H. erinaceus* strains. We predicted the genes related to the biosynthetic pathway of bioactive compounds, especially polysaccharides. The key enzymes involved in polysaccharide biosynthesis, such as PGM, PGI and UGP, were identified, and the expression levels of these genes were relatively consistent with the polysaccharide contents in the six strains. Moreover, the genes and gene clusters related to the saccharide unit were detected. The GO analysis of transcript and functional annotation revealed that signal transduction had the most hits, followed by carbohydrate metabolism, translation, transport and catabolism, and amino acid metabolism. Furthermore, we performed ORF and SSR detection. These datasets will be valuable for the biotechnology industry in the production and packaging of these metabolites for commercial applications of mushrooms in the future and will improve our knowledge of *H. erinaceus* biology.

## Additional files

**Additional file 1: Figure S1.** The distribution of the base quality in six strains sequenced data. A. *H. erinaceus* HT4903, B. *H. erinaceus* GT-06, C. *H. erinaceus* CC-02, D. *H. erinaceus* PZH-05, E. *H. erinaceus* TJH-03, F. *H. erinaceus* TD-04. **Figure S2.** The distribution of A, T, G, C composition in six strains. A. *H. erinaceus* HT4903, B. *H. erinaceus* GT-06, C. *H. erinaceus* CC-02, D. *H. erinaceus* PZH-05, E. *H. erinaceus* TJH-03, F. *H. erinaceus* TD-04. **Figure S3**. The distribution of genes FPKM in six strains. A. *H. erinaceus* HT4903, B. *Hericium erinaceus* GT-06, C. *H. erinaceus* CC-02, D. *H. erinaceus* PZH-05, E. *H. erinaceus* TJH-03, F. *H. erinaceus* TD-04. **Figure S4.** The distribution of transcripts FPKM in 6 strains. A. *H. erinaceus* HT4903, B. *Hericium erinaceus* GT-06, C. *H. erinaceus* CC-02, D. *H. erinaceus* PZH-05, E. *H. erinaceus* TJH-03, F. *H. erinaceus* TD-04. **Table S1.** The morphological characteristic of six strains in *H.erinaceus.* **Table S2.** Statistics of the genes and transcripts number in different expression levels. **Table S3.** The number of genes enriched to the KEGG pathway.

**Additional file 2: Dataset 1.** The specific primers of 10 genes selected to qRT-PCR experiment.

**Additional file 3: Dataset 2.** *H. erinaceus*-FPKM top10 transcript.

**Additional file 4: Dataset 3.** 13-co-existed genes involved in polysaccharides biosynthesis and 5 genes involved in fructose and mannose metabolism.

**Additional file 5: Dataset 4.** The annotated of all transcripts in six *H.erinaceus* samples.

## Data Availability

The datasets generated and analyzed during the current study are available in the NCBI repository (accession number PRJNA503307).
