# Supplementary material for: Comparative transcriptome analysis reveals the genetic basis underlying the biosynthesis of polysaccharides in Hericium erinaceus
Source: Bot Stud. 2019 Jul 30;60:15. doi: 10.1186/s40529-019-0263-0 (PMC6667577; doi:10.1186/s40529-019-0263-0)
Supplement: Supplementary file 1 — Additional file 1: Figure S1. The distribution of the base quality in six strains sequenced data. A. H. erinaceus HT4903, B. H. erinaceus GT-06, C. H. erinaceus CC-02, D. H. erinaceus PZH-05, E. H. erinaceus TJH-03, F. H. erinaceus TD-04. Figure S2. The distribution of A, T, G, C composition in six strains. A. H. erinaceus HT4903, B. H. erinaceus GT-06, C. H. erinaceus CC-02, D. H. erinaceus PZH-05, E. H. erinaceus TJH-03, F. H. erinaceus TD-04. Figure S3. The distribution of genes FPKM in six strains. A. H. erinaceus HT4903, B. Hericium erinaceus GT-06, C. H. erinaceus CC-02, D. H. erinaceus PZH-05, E. H. erinaceus TJH-03, F. H. erinaceus TD-04. Figure S4. The distribution of transcripts FPKM in 6 strains. A. H. erinaceus HT4903, B. Hericium erinaceus GT-06, C. H. erinaceus CC-02, D. H. erinaceus PZH-05, E. H. erinaceus TJH-03, F. H. erinaceus TD-04. Table S1. The morphological characteristic of six strains in H.erinaceus. Table S2. Statistics of the genes and transcripts number in different expression levels. Table S3. The number of genes enriched to the KEGG pathway. [file 40529_2019_263_MOESM1_ESM.pdf]

Comparative transcriptome analysis reveals the genetic basis underlying the biosynthesis of Polysaccharide in *Hericium erinaceus*

Nan Zhang1, 2, Zongfu Tang1, Jun Zhang1, Xin Li1, ZiqianYang1, Chun Yang1, Zhaofeng Zhang1, Zuoxi Huang1

Nan Zhang: emailzhangnan@njtc.edu.cn; Zongfu Tang: email15760669307@163.com; Jun Zhang: email2069677203@qq.com; Xin Li: email15982744079@163.com; Ziqian Yang: email1466252383@qq.com; Chun Yang: email1925542442@qq.com; Zhaofeng Zhang: email15378440748@163.com.

Corresponding author E-mail address: huangzx118@126.com

1 College of Life Sciences, Neijiang Normal University, Neijiang, 641100, PR China

2 Key Laboratory of Regional Characteristic Agricultural Resources, Department of Education, Neijiang, 641100, PR China

Supplementary Information Figures

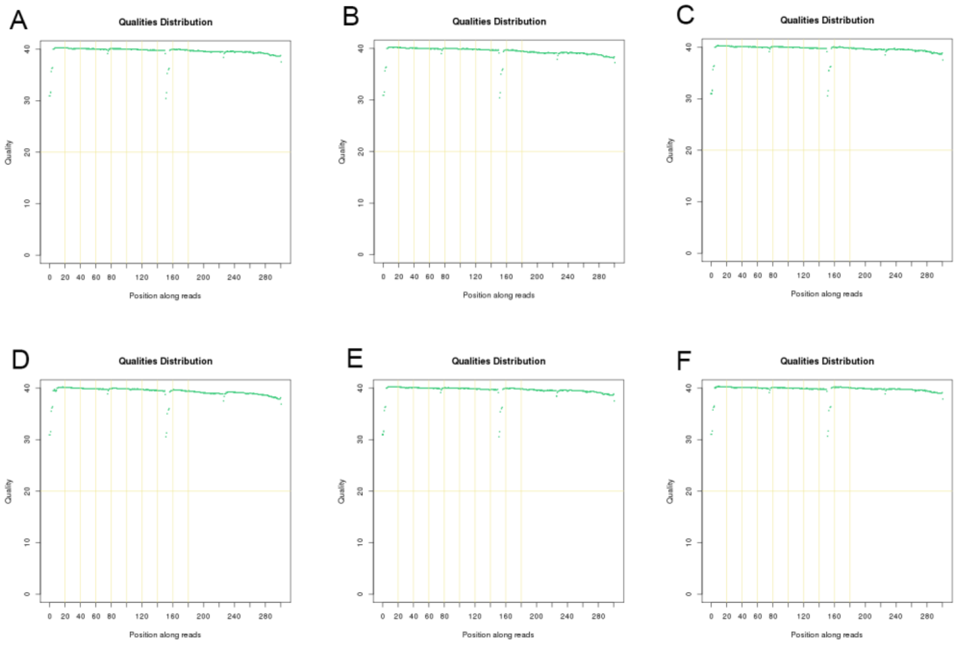

Figure S1. The distribution of the base quality in six strains sequenced data. A. *H. erinaceus* HT4903, B. *H. erinaceus* GT-06, C. *H. erinaceus* CC-02, D. *H. erinaceus* PZH-05, E. *H. erinaceus* TJH-03, F. *H. erinaceus* TD-04.

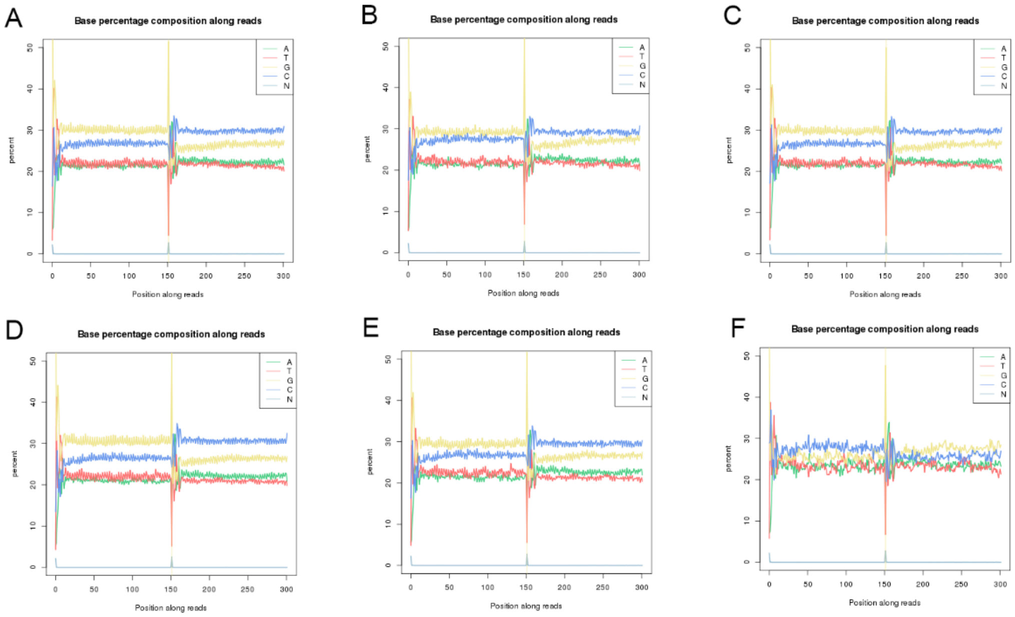

Figure S2. The distribution of A, T, G, C composition in six strains. A. *H. erinaceus* HT4903, B. *H. erinaceus* GT-06, C. *H. erinaceus* CC-02, D. *H. erinaceus* PZH-05, E. *H. erinaceus* TJH-03, F. *H. erinaceus* TD-04.

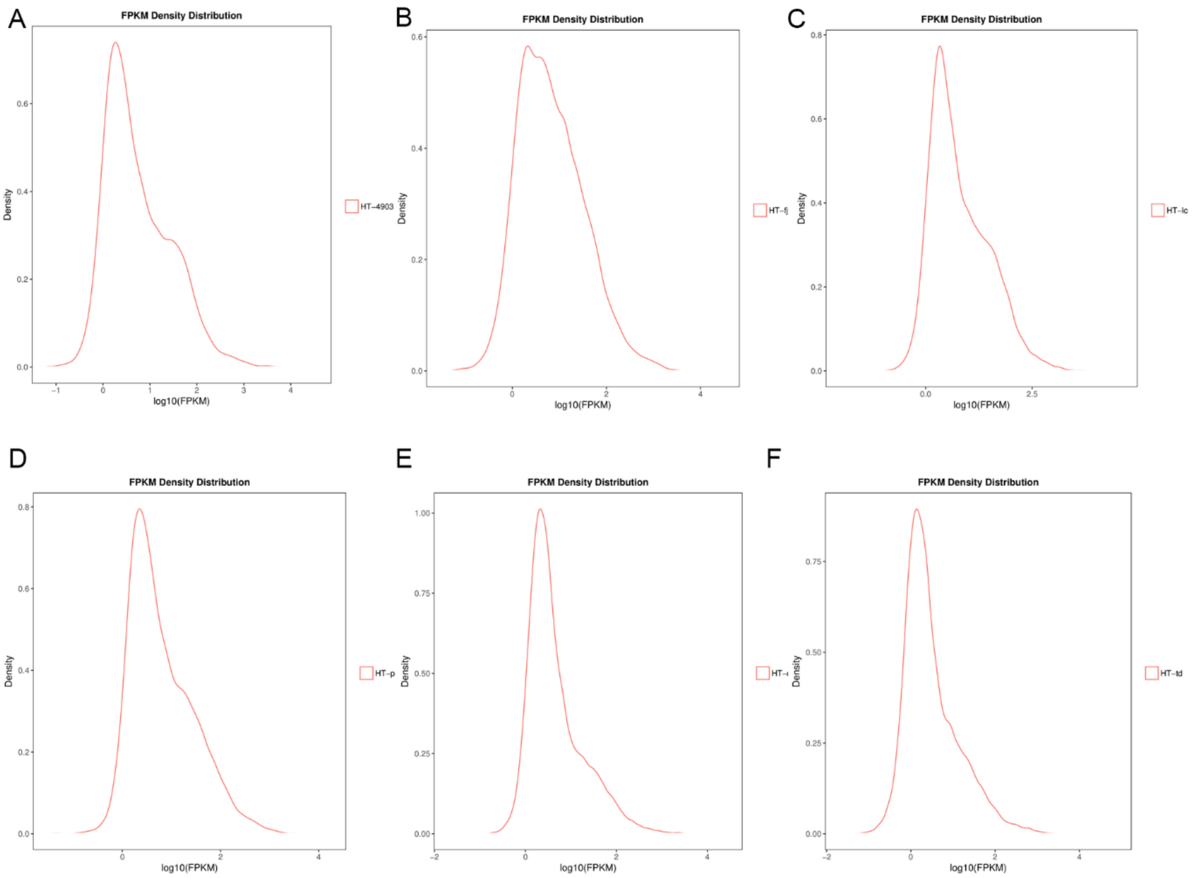

Figure S3. The distribution of genes FPKM in six strains. A. *H. erinaceus* HT4903, B. *Hericium erinaceus* GT-06, C. *H. erinaceus* CC-02, D. *H. erinaceus* PZH-05, E. *H. erinaceus* TJH-03, F. *H. erinaceus* TD-04.

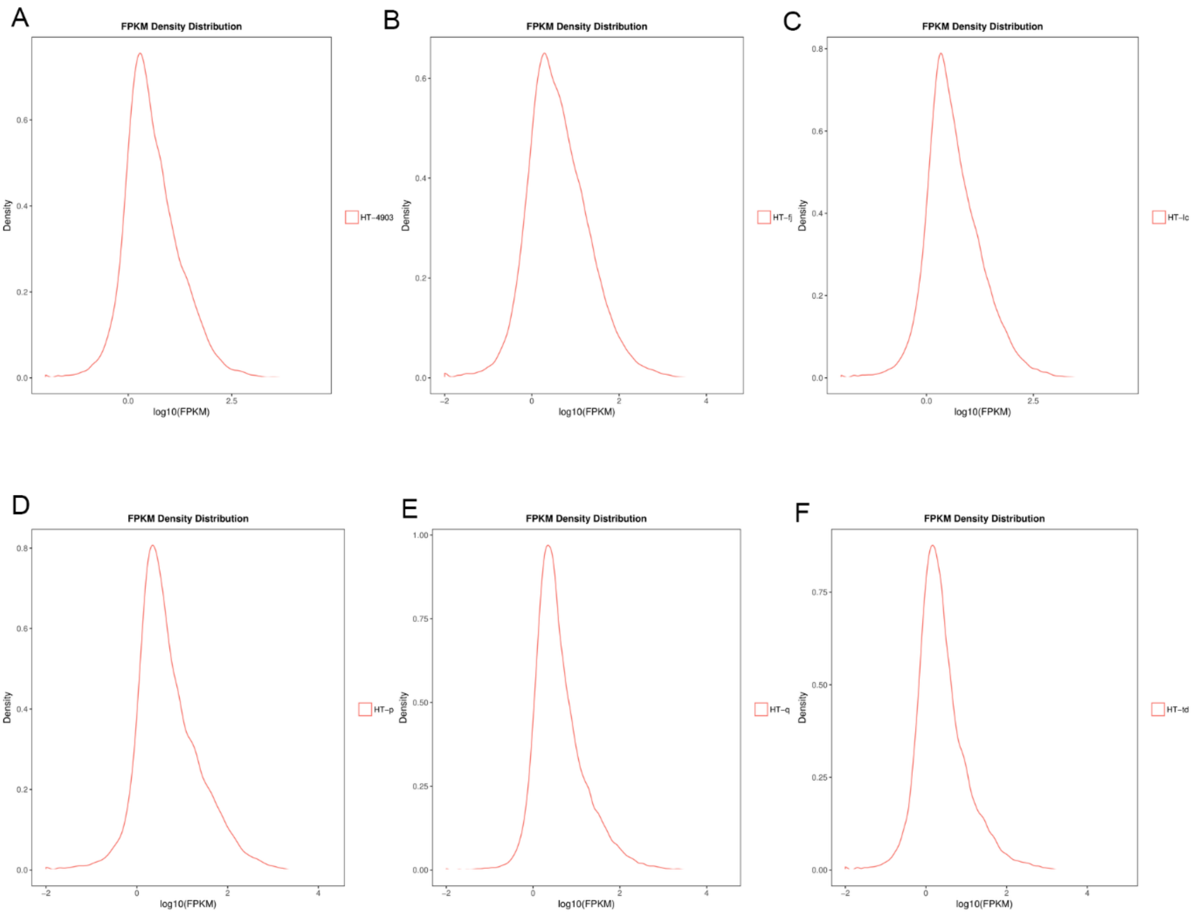

Figure S4. The distribution of transcripts FPKM in 6 strains. A. *H. erinaceus* HT4903, B. *Hericium erinaceus* GT-06, C. *H. erinaceus* CC-02, D. *H. erinaceus* PZH-05, E. *H. erinaceus* TJH-03, F. *H. erinaceus* TD-04.

Supplementary Information Tables

Table S1. The morphological characteristic of six strains in *H.erinaceus*.

Sample	Mycelia characteristic	Growth state	Fruiting body	Growing rate
***H.erinaceus* HT-4903**	yellowish, dense and irregular mycelia edges	The growth is slow	The spines of aerial mycelia are short and dense, the fruiting body are medium	12-14 days to overgrow across culture medium with diameter of 90mm, and the daily growth rate of mycelia is 5 mm.
***H.erinaceus* GT-06**	white, thick, dense and irregular mycelia edges	Grows slowly and adherent on the medium, creeping and spreading close to the surface of the medium	The spines of aerial mycelia are short and dense, the fruiting body are strong	11-12 days to overgrow across culture medium with diameter of 90mm, and the daily growth rate of mycelia is 7 mm.
***H.erinaceus* CC-02**	yellowish, sparse and scattered, with irregular mycelia edges	Growing slow	The spines of aerial mycelia are long, the fruiting body are strong	15-16 days to overgrow across culture medium with diameter of 90mm, and the daily growth rate of mycelia is 4 mm.
***H.erinaceus* PZH–05**	powdery white, radial, thin, dense, and neat mycelia edges	Growth is extremely fast, and adherent growth	no aerial mycelia	3 days to overgrow across culture medium with diameter of 90mm, and the daily growth rate of mycelia is 23 mm.
***H.erinaceus* TJH–03**	cream-colored yellowish, close to white, dense and fluffy, with irregular mycelia edges	Growing fast, spreading and creeping radially from the inoculation point to the surrounding area	Aerial mycelia is strong, and the color is milk white.	5-6 days to overgrow across culture medium with diameter of 90mm, and the daily growth rate of mycelia is 12 mm.
***H.erinaceus* TD–04**	brown, sparse, with irregular mycelia edges	Growth relatively slow	The spines of aerial mycelia are long, the fruiting body are strong	11-12 days to overgrow across culture medium with diameter of 90mm, and the daily growth rate of mycelia is 10 mm.

Table S2. Statistics of the genes and transcripts number in different expression levels.

Statistics of the genes number in different expression levels						
**Sample**	**0**	**0~1**	**1~3**	**3~15**	**15~60**	**>60**
**HT-4903**	1,363(6.03%)	2,194(9.70%)	7,048(31.16%)	6,440(28.47%)	3,603(15.93%)	1,970(8.71%)
**GT-06**	3,213(12.90%)	2,024(8.12%)	5,553(22.29%)	7,749(31.10%)	4,210(16.90%)	2,166(8.69%)
**CC-02**	1,118(5.02%)	1,566(7.03%)	6,806(30.54%)	6,893(30.93%)	3,776(16.94%)	2,125(9.54%)
**PZH-05**	933(4.46%)	1,060(5.07%)	6,531(31.25%)	7,008(33.53%)	3,499(16.74%)	1,871(8.95%)
**TJH-03**	2,441(6.55%)	2,536(6.81%)	14,407(38.67%)	11,502(30.87%)	4,219(11.32%)	2,154(5.78%)
**TD-04**	1,367(4.77%)	5,752(20.08%)	10,734(37.48%)	6,639(23.18%)	2,840(9.92%)	1,308(4.57%)
**Statistics of the transcripts number in different expression levels**						
**Sample**	**0**	**0~1**	**1~3**	**3~15**	**15~60**	**>60**
**HT-4903**	2,800(7.58%)	5,365(14.52%)	11,459(31.02%)	11,305(30.60%)	4,293(11.62%)	1,723(4.66%)
**GT-06**	2,486(6.89%)	3,983(11.04%)	10,987(30.46%)	12,157(33.71%)	4,586(12.72%)	1,866(5.17%)
**CC-02**	3,169(6.70%)	3,849(8.14%)	17,494(36.99%)	16,130(34.11%)	4,732(10.01%)	1,920(4.06%)
**PZH-05**	5,014(12.49%)	6,005(14.96%)	10,294(25.64%)	11,901(29.65%)	4,903(12.21%)	2,024(5.04%)
**TJH-03**	1,502(5.80%)	2,400(9.26%)	8,186(31.60%)	8,572(33.09%)	3,452(13.33%)	1,793(6.92%)
**TD-04**	2,481(6.11%)	9,228(22.73%)	14,908(36.73%)	9,878(24.34%)	2,901(7.15%)	1,194(2.94%)

Table S3. The number of genes enriched to the KEGG pathway.

KEGG_ID	Number of genes (remove duplicate)					
	**HT-4903**	**GT-06**	**CC-02**	**PZH-05**	**TJH-03**	**TD-04**
**ko00500**	167 (59)	173 (69)	148 (65)	92 (43)	185 (94)	142 (64)
**ko00051**	65 (34)	85 (38)	58 (31)	51 (18)	86 (59)	71 (41)
**ko00052**	60 (26)	59 (29)	51 (23)	33 (18)	67 (43)	63 (26)
**Total (remove duplicate)**	282 (97)	317 (113)	257 (97)	176 (66)	338 (162)	276 (111)
